# The autophagy–lysosomal system in subarachnoid haemorrhage

**DOI:** 10.1111/jcmm.12855

**Published:** 2016-03-29

**Authors:** Haijian Wu, Huanjiang Niu, Cheng Wu, Yong Li, Kun Wang, Jianmin Zhang, Yirong Wang, Shuxu Yang

**Affiliations:** ^1^ Department of NeurosurgerySir Run Run Shaw HospitalSchool of MedicineZhejiang UniversityHangzhouChina; ^2^ Department of NeurosurgerySchool of MedicineNingbo UniversityNingboChina; ^3^ Department of NeurosurgerySecond Affiliated HospitalSchool of MedicineZhejiang UniversityHangzhouChina

**Keywords:** autophagy, lysosome, cell death, neuroprotection, subarachnoid haemorrhage

## Abstract

The autophagy–lysosomal pathway is a self‐catabolic process by which dysfunctional or unnecessary intracellular components are degraded by lysosomal enzymes. Proper function of this pathway is critical for maintaining cell homeostasis and survival. Subarachnoid haemorrhage (SAH) is one of the most devastating forms of stroke. Multiple pathogenic mechanisms, such as inflammation, apoptosis, and oxidative stress, are all responsible for brain injury and poor outcome after SAH. Most recently, accumulating evidence has demonstrated that the autophagy–lysosomal pathway plays a crucial role in the pathophysiological process after SAH. Appropriate activity of autophagy–lysosomal pathway acts as a pro‐survival mechanism in SAH, while excessive self‐digestion results in cell death after SAH. Consequently, in this review article, we will give an overview of the pathophysiological roles of autophagy–lysosomal pathway in the pathogenesis of SAH. And approaching the molecular mechanisms underlying this pathway in SAH pathology is anticipated, which may ultimately allow development of effective therapeutic strategies for SAH patients through regulating the autophagy–lysosomal machinery.

## Introduction

The autophagy–lysosomal system is a self‐destructive process by which cytoplasmic substrates are delivered to lysosomes for degradation 1. It serves as an important homeostatic mechanism that is responsible for clearance of damaged organelles and protein aggregates in eukaryotic cells 2. Dysfunction of autophagy–lysosomal pathway has been linked to various disease states, such as cancers, infectious diseases and neurodegenerative diseases 3, 4, 5, 6. Thus, deciphering molecular mechanisms of this degradation pathway would contribute to harness this process for therapeutic purposes.

Subarachnoid haemorrhage (SAH) is a serious, life‐threatening type of stroke, which denotes the presence of blood within the subarachnoid space 7. Approximately 85% of cases of spontaneous SAH occur from the rupture of intracranial aneurysms, 10% fit into the pattern of peri‐mesencephalic non‐aneurysmal haemorrhages and the remaining 5% are caused by other medical conditions, such as inflammatory or non‐inflammatory lesions of intracerebral vessels, sickle cell disease, coagulopathies, neoplasms or drugs 8. It is noteworthy that although SAH accounts for only 5% of all strokes, its burden on individuals, their families and society is significant, because of high mortality and disability rates, and remarkable incidence among young adults 9. On the other hand, despite considerable advances in diagnosis and treatment of SAH, clinical outcome remains disappointing and effective therapeutic strategies are yet to be established 9. As a consequence, further improving the understanding of SAH pathophysiology is emphasized.

Currently, early brain injury (EBI) and delayed brain injury (DBI) are conceived as two most important mechanisms for SAH pathology 10. Experimental evidence has demonstrated that the function of subcellular organelles is altered and is implicated in the pathogenesis of brain injury after SAH 11. Molecular events, such as transcription factor translocation, endoplasmic reticulum stress and mitochondrial dysfunction, occur in the neurovascular unit after SAH 12, 13, 14. More importantly, the ‘self‐eating’ autophagy–lysosomal cascades are activated and play an important role in the pathophysiology of SAH 15. Thus, this review aims to survey the role and underlying mechanism of autophagy–lysosomal system in the pathogenesis of EBI and DBI after SAH, which may ultimately contribute to develop novel therapeutic targets for SAH treatment *via* modulating this pathway.

## The mechanism and regulation of autophagy–lysosomal system

**Figure 1:**
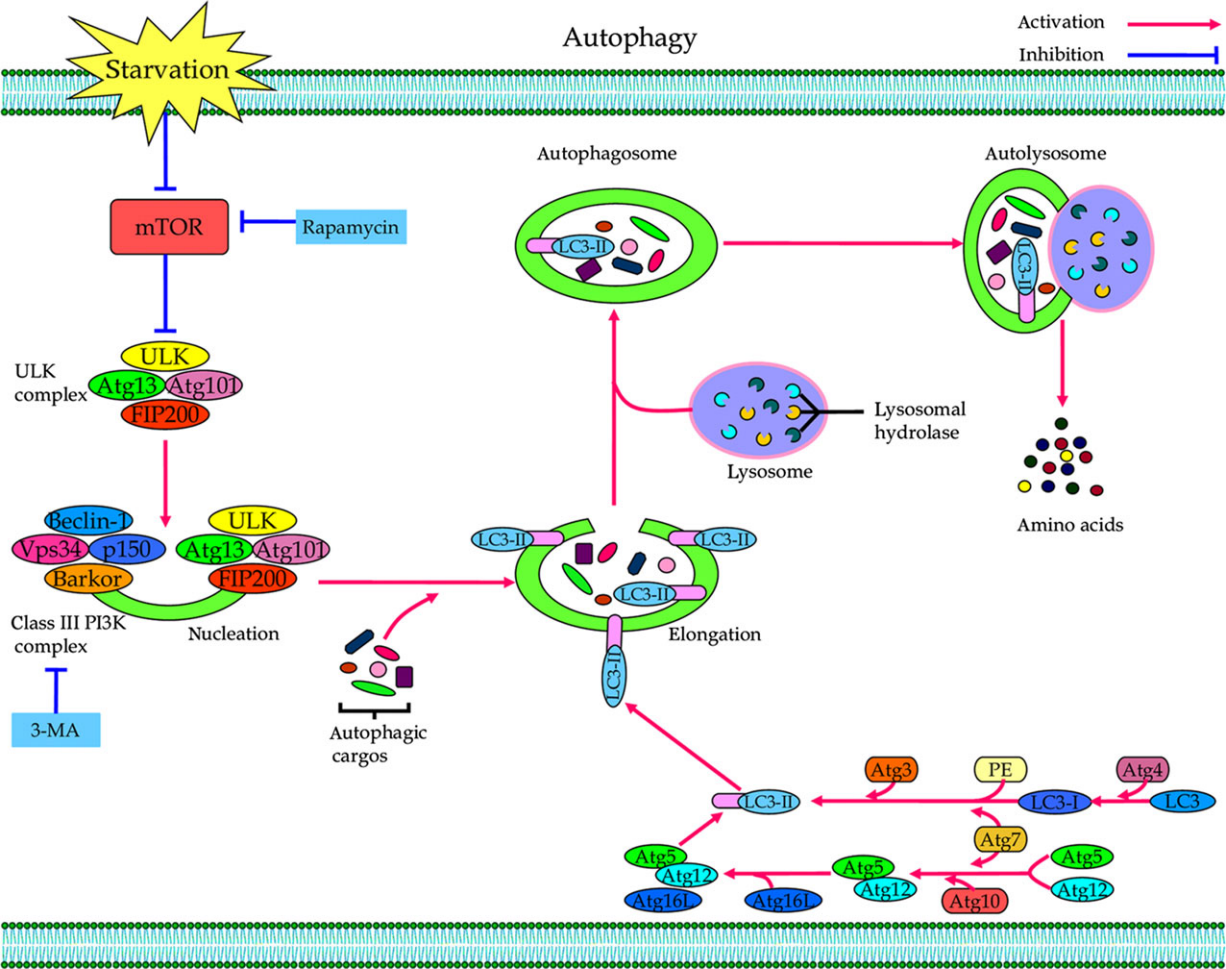
Overview of the cellular and molecular events of autophagy–lysosomal pathway. The autophagy–lysosomal process consists of a series of sequential steps: nucleation, elongation, maturation, docking, fusion and degradation. Several hetero‐oligomeric protein complexes that contain autophagy‐related (Atg) proteins play a critical role at different stages of autophagy–lysosomal process. Multiple signalling pathways, including mTOR dependent and independent, participate in the regulation of autophagy–lysosomal cascades in response to numerous environmental and cellular stimuli.

Autophagy is a sophisticated catabolic process in which cytosolic components and organelles are transported to lysosomes for degradation 16. Depending on the mode of cargo delivery to lysosome, autophagy is commonly divided into three main subtypes, namely microautophagy, chaperone‐mediated autophagy (CMA) and macroautophagy 17, 18, 19. In this review, we will focus on macroautophagy (hereafter referred to as autophagy), the major type of autophagy–lysosomal pathway that eukaryotic cells use to degrade long‐lived proteins and organelles (Fig. 1) 20. In the case of this autophagic process, cytoplasmic cargos are sequestered into double‐membrane vesicles known as autophagosomes, which are then delivered to the lysosomes for degradation 21. Mechanistically, the autophagy–lysosomal pathway can be broken down into series of sequential steps: nucleation, elongation, maturation, docking, fusion and degradation 22. In detail, it begins with initiation and nucleation, where cup‐shaped membrane structures termed phagophores are formed 23. Then, portions of cytoplasm, including organelles, are enclosed by phagophores to form autophagosomes 24. Autophagosomes are thereafter trafficked to the lysosomes to form autolysosomes, where the captured substrates, together with the inner membrane, are degraded by lysosomal enzymes 25. The resulting monomeric units (*e.g*. amino acids) are subsequently exported to the cytoplasm for reuse.

Importantly, several hetero‐oligomeric protein complexes that contain autophagy‐related (Atg) proteins exert a critical role at different stages of autophagy 26. As an example, the Unc‐51 like autophagy activating kinase (ULK) complex, consisting of ULK1/2, Atg13, FIP200 (focal adhesion kinase family–interacting protein of 200 kD) and Atg101, is essential for the initiation of autophagy 27. Under normal nutritional conditions, the serine/threonine kinase mammalian target of rapamycin (mTOR) complex 1 targets the ULK complex and inactivates it by phosphorylation of ULK1/2 and Atg13 28. Kim *et al*. reported that high mTOR activity prevents ULK1 activation by phosphorylating ULK1 Ser 757 when nutrients are plentiful 29. In contrast, when nutrients are depleted, the mTOR activity is inhibited and phosphorylation of ULK1 Ser 757 is decreased, and subsequently ULK1 can interact with and be phosphorylated by AMP‐activated protein kinase (AMPK) on Ser 317 and Ser 777, which resulting in activation of ULK1 kinase and autophagy induction 27, 29. This evidence indicates that different phosphorylation events have distinct functions in autophagy initiation. Downstream of the ULK complex, the class III phosphatidylinositol 3‐kinase (PI3K) complex, composed of class III PI3K, Beclin‐1, p150 and barkor (Beclin‐1‐associated autophagy‐related key regulator), is required for the nucleation and assembly of the initial phagophore membrane 30, 31, 32. The elongation and closure of phagophores depends on two ubiquitination‐like reactions. In the first of the reactions, the ubiquitin‐like protein Atg12 is covalently tagged to Atg5 to form Atg12–Atg5 conjugate, with the help of the E1‐like enzyme Atg7 and the E2‐like enzyme Atg10 33. The Atg12–Atg5 then conjugates with Atg16L (Atg16‐like protein) to form an ~800‐kDa protein complex, which serves as a platform for stimulating the microtubule‐associated protein 1 light‐chain 3 (LC3)‐PE (phosphatidylethanolamine) conjugation 34, 35. In the second ubiquitin‐like reaction, the precursor LC3 is cleaved at its COOH terminus by the protease Atg4B, resulting in the cytosolic isoform LC3‐I. LC3‐I is conjugated to PE to form LC3‐II with the action of the E1‐like enzyme Atg7 and the E2‐like enzyme Atg3 36, 37. Thus, the conversion of LC3‐I to LC3‐II is a well‐known marker of autophagy induction 38. More importantly, the lipidated form of LC3, namely LC3‐II, mediates membrane tethering and hemifusion that essential for the expansion and closure of phagophores to form autophagosomes during autophagy 39.

Because of its pathophysiological significance in cellular self‐cannibalism, the autophagic process must be tightly regulated. In mammalian cells, multiple signalling cascades, including mTOR‐dependent and mTOR‐independent pathways, participate in the regulation of autophagy in response to numerous environmental and cellular stimuli 40. As aforementioned above, the classical mTOR pathway acts as a major negative regulator of autophagy through blocking the ULK complex 29, 41. Apart from the classical mTOR pathway, the mTOR‐independent pathways, such as the cAMP‐Epac‐phospholipase C (PLC)‐ε‐inositol 1,4,5‐trisphosphate (IP_3_) pathway and the Ca^2+^‐calpain‐G‐stimulatory protein α (G_s_α) pathway, can also regulate autophagy in mammalian systems 42, 43. As an example, elevation of intracellular cAMP levels by adenylate cyclase (AC) activates Epac, and activated Epac in turn activates a small G protein Rap2B, leading to PLC‐ε‐mediated hydrolysis of phosphatidylinositol 4,5‐bisphosphate (PIP2) to generate IP3, which eventually inhibits autophagy 44. Besides, an increase in cytosolic Ca^2+^ activates calpains, and activated calpain activates Gsα, resulting in enhanced AC activity that generates cAMP to suppress autophagy 43. Additionally, starvation‐induced activation of c‐Jun N‐terminal protein kinase 1 phosphorylates Bcl‐2, which allows Bcl‐2 to dissociate from the autophagy‐inhibitory Beclin‐1–Bcl‐2 complex, thereby promoting the formation of the autophagy‐initiating Beclin‐1–Vps34 complex to drive autophagy 45, 46. In contrast, molecular mechanisms underlying the processes of autophagosome transport, autophagosome–lysosome fusion, autolysosomal degradation and reutilization of degradation products, are just beginning to be understood and warranted to be further investigated.

## The autophagy–lysosomal system in subarachnoid haemorrhage: potential targets for therapeutic intervention

**Figure 2:**
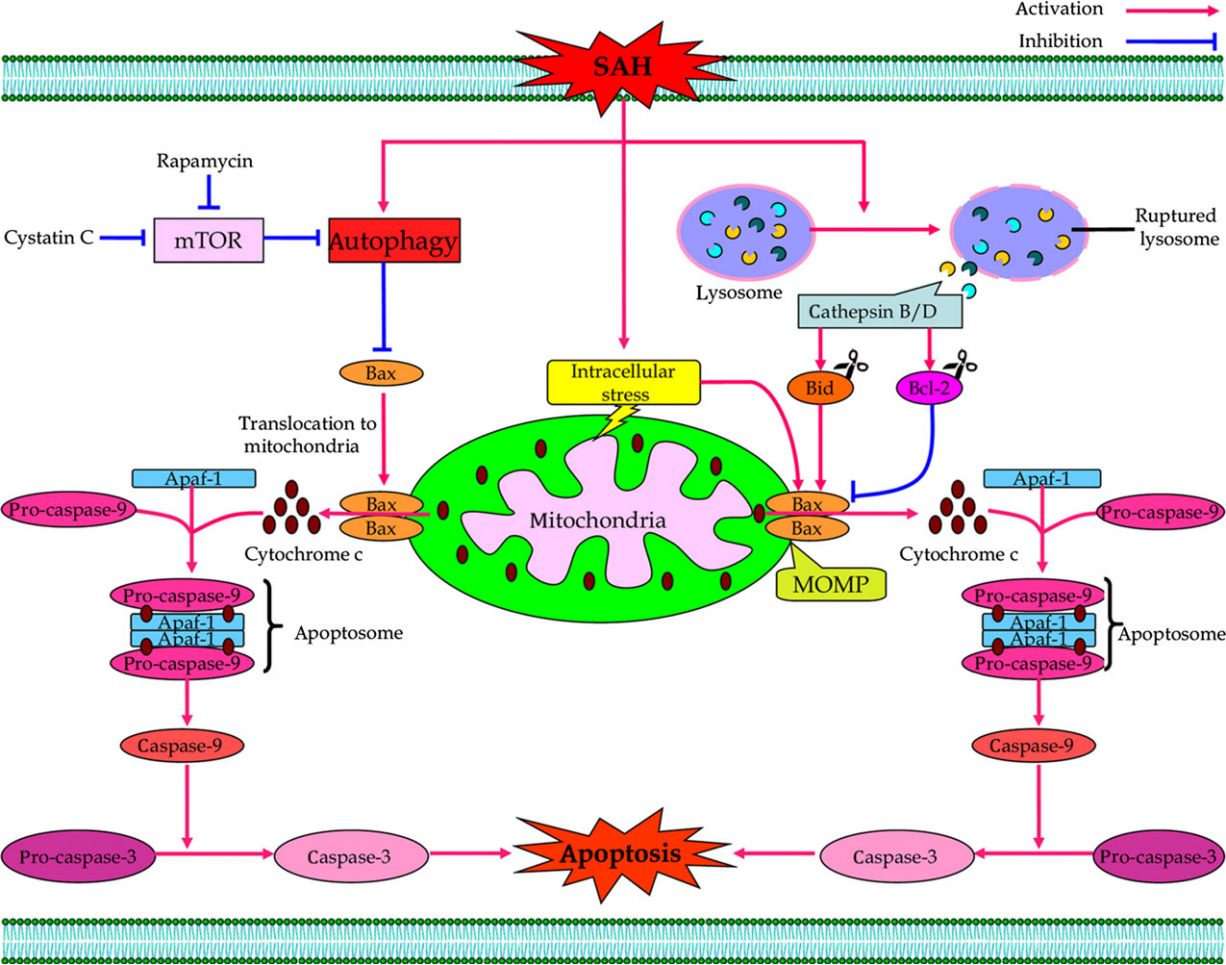
Scheme of the role of autophagy–lysosomal system in the pathophysiology of SAH. The autophagy–lysosomal pathway plays a vital role in the pathophysiological process of SAH. Appropriate autophagy–lysosomal activity acts as a pro‐survival mechanism in SAH, while excessive self‐digestion of autophagy results in cell death after SAH.

Subarachnoid haemorrhage is a complex, multisystem and multifaceted disorder which involves several ongoing pathological processes 47. EBI and DBI have been recognized as the important determinants of morbidity and mortality as well as worsened clinical outcome for SAH patients 48. EBI was coined to describe the acute pathophysiological events occurring in the brain within the first 72 hrs after an SAH 49. Early pathological changes, such as acute global ischaemia, mechanical and biochemical alterations, impaired ionic homeostasis, excitotoxicity, oxidative stress, inflammation and apoptosis, are all clinically relevant to the poor outcome of SAH patients 50. By contrast, DBI is designated to demonstrate a host of critical, interrelated pathological events arising in the late phase (3–14 days) of SAH 48. Cerebral vasospasm (CVS) is conceived as a major cause of delayed cerebral ischaemia and plays a crucial role in the pathogenesis of DBI following SAH 51. It is noteworthy that molecular mechanisms leading to EBI and DBI are not mutually exclusive. Instead, multiple pathological pathways deleterious to brain activate after the initial haemorrhage, evolve with time and eventually contribute to overall outcome of SAH. More importantly, the autophagy–lysosomal pathway is activated and involved in the pathophysiologic process of SAH (Fig. 2). In consideration of the importance of autophagy–lysosomal system for neuronal survival, its pathological significance and underlying mechanisms are discussed below.

### Autophagy and brain injury

The autophagy–lysosomal system is a catabolic process that allows the degradation and recycling of intracellular components to ensure cell homeostasis and survival. In a modified endovascular perforation rat model of SAH, Lee *et al*. demonstrated that the autophagy–lysosomal pathway is activated in the ipsilateral frontobasal cortex following SAH and lasts during the entire phase of EBI (up to 3 days) 15. For electron microscopy, numerous double‐ or multiple‐membrane autophagic vesicles are predominantly observed in neurons at 24 hrs following SAH, demonstrating enhanced autophagy–lysosomal pathway activity in neuronal cells after SAH 15. By applying Rapamycin (an autophagy inducer targeting mTOR) or 3‐Methyladenine (3‐MA) (an autophagy inhibitor) to manipulate the autophagic activity, the potential beneficial effect of autophagy on EBI was examined in rat endovascular perforation models of SAH 52, 53. Autophagy activation reduces translocation of Bax, a pro‐apoptotic member of Bcl‐2 family, from the cytosol to the mitochondrial membrane 52. As a consequence, Bax‐mediated mitochondrial outer membrane permeabilization is alleviated, the subsequent cytochrome c release into the cytosol is decreased, and the mitochondrial apoptotic pathway is eventually inhibited 54. Interestingly, melatonin, a hormone secreted by the pineal gland in the brain, stimulated autophagy to suppress apoptotic death of neural cells and ameliorated neurological deficits after SAH 55. The anti‐apoptotic effect of melatonin‐enhanced autophagy is associated with prevention of mitochondrial release of cytochrome c to mediate caspase‐dependent apoptotic cascades 55. Also, Shao *et al*. demonstrated that trichostatin A, a pan‐histone deacetylase inhibitor, significantly increased the levels of Beclin‐1 and LC3‐II/LC3‐I ratio, while decreased the expression of Bax and cleaved caspase‐3 in the cortex at 24 hrs after experimental SAH 56. Simultaneously, SAH‐induced neuronal apoptosis in the ipsilateral basal cortex was significantly inhibited, and neurological deficits are largely attenuated after TSA application 56. However, it remains to be investigated whether TSA and other histone deacetylase inhibitors could correct potentially low levels of histone acetylation status following SAH, thus facilitate Atg genes transcription through increasing the accessibility of their promoters to related transcription factors 57. Additionally, autophagy activation in response to endoplasmic reticulum stress is protective in prevention of EBI in the rat endovascular perforation SAH model 58. Inhibition of autophagy with 3‐MA promoted apoptotic cascades, aggravated neurological deficits and naturalized the endoplasmic reticulum stress‐induced beneficial effect after SAH 58.

Similar to the data from endovascular perforation SAH models, Wang *et al*. found that the expression of LC3 and Beclin‐1 was significantly increased in the cortex and peaked at 24 hrs after prechiasmatic cistern blood injection, indicating the activation of autophagy–lysosomal system in the brain post‐SAH 59. The autophagy activator Rapamycin can up‐regulate the expressions of LC3 and Beclin‐1, down‐regulate cortical apoptosis, ameliorate blood–brain barrier (BBB) permeability and alleviate clinical behaviour function impairment caused by SAH 59. Conversely, the autophagy inhibitor 3‐MA can decrease the expressions of LC3 and Beclin‐1, increase cortical apoptosis, promote BBB permeability and ultimately aggravate clinical behaviour function impairment induced by SAH 59. Liu *et al*. demonstrated that pre‐treatment with a cysteine protease inhibitor Cystatin C with low or medial dosages promotes the autophagic process within neurons, inhibits BBB impairment and ameliorates brain oedema formation, which contributes to alleviation of secondary learning deficits in the prechiasmatic cistern SAH injection model 60. Cystatin C‐mediated inhibition of mTOR‐signalling pathway may contribute to autophagy activation under those stress conditions 61, 62. Taken together, these findings suggested the protective contribution of autophagy–lysosomal pathway in the pathogenesis of EBI during experimental SAH.

### Autophagy and cerebral vasospasm

Cerebral vasospasm is one of the most common and devastating sequelae for patients who have sustained SAH. It is involved in the development of delayed cerebral ischaemia and contributes to DBI after SAH 51. The contribution of autophagy–lysosomal system in the pathogenesis of CVS following SAH has also been investigated. In a rat cisterna magna single‐injection model of SAH, Liu *et al*. demonstrated that the autophagic pathway is activated in the spastic basilar arteries after SAH 63. Interestingly, Cystatin C promotes the activation of autophagy in the walls of basilar arteries and ameliorates the degree of CVS in this SAH model 63. However, the exact anti‐vasospasm mechanisms of autophagy remain unknown and are warranted to be clarified.

### Lysosome and cathepsin in subarachnoid haemorrhage

Lysosomes, the cytoplasmic membrane‐enclosed organelles that contain hydrolytic enzymes, are the key degradative compartments of the cell that control the intracellular turnover of macromolecules 64. The lysosomal hydrolases including cathepsins, which are enclosed in the lysosomes, play a crucial role in the degradation of heterophagic and autophagic material 65. It is important to note that lysosomes participate in cellular iron metabolism and recycling 66. Because of this, most lysosomes contain relatively large amounts of redox‐active iron 67. These iron‐rich lysosomes are unusually susceptible to destabilization in response to oxidative challenge, resulting in the release of hydrolytic enzymes (*i.e*. cathepsin B/D) into the cytoplasm, which in turn trigger the lysosomal pathway of apoptosis through cleavage of the pro‐apoptotic Bcl‐2 family member Bid and the degradation of the anti‐apoptotic Bcl‐2 members such as Bcl‐2, Bcl‐xL and Mcl‐1 68.

In the setting of SAH, lysosomes may become iron overload and is particularly prone to destabilization, resulting in lysosomal membrane rupture and the release of hydrolytic enzymes into cytoplasm 69, 70. Alternatively, overactivation of autophagy may lead to the accumulation of enlarged and unstable acidic vesicles, which would contribute to lysosomal permeabilization and hydrolytic enzymes released from destabilized autolysosomes 71, 72. However, whether these events are key mediators in SAH‐induced hydrolytic enzymes up‐regulation deserve further investigation. During the acute phase of SAH, the levels of cathepsin B/D and caspase‐3 were up‐regulated in the neuron of rat cortex soon after blood injection, which peaked at 48 hrs post‐SAH, suggested that the lysosomal membrane of neuron was damaged after SAH 70. The disruption of lysosomal membrane allows lysosomal proteases (*i.e*. cathepsin B/D) to be released into the cytoplasm to activate caspase‐dependent apoptotic pathway 70, 73, 74. Intraperitoneal administration of deferoxamine, an iron chelator, down‐regulates expression of cathepsin B/D and prevents up‐regulation of caspase‐3 in the cortex 48 hrs after SAH, which contributes to attenuate apoptotic cell death, BBB permeability, brain oedema and motor deficits after SAH 70. More recently, Wang *et al*. also demonstrated that lysosomes were impaired and cathepsin B/D was up‐regulated in the cerebral cortex of affected rats under SAH conditions 75. By contrast, α‐lipoic acid‐plus, an amine derivative of α‐lipoic acid, can provide neuroprotective effects against EBI *via* targeting lysosomes and chelating intra‐lysosomal iron in this prechiasmatic cistern SAH model 75. Treatment with α‐lipoic acid‐plus reduces oxidative stress and decreases iron deposition in the cortex of brain, alleviates lysosomal membrane permeabilization and prevents lysosomal rupture following SAH 75, 76. As a result, the protein levels of cathepsin B/D in the cytoplasm of neurons are decreased and the ensuing Bax‐induced apoptotic cell death is reduced, which is protective for amelioration of BBB disruption, brain oedema and neurological behaviour impairment after experimental SAH 75, 77.

Additionally, an imbalance between cysteine cathepsin enzymes and their inhibitor Cystatin C in the arterial walls may exert a prominent role in the progression and rupture of cerebral aneurysms 78, 79. When compared with the control cerebral arterial walls, cathepsin B, K and S were highly expressed in the intima and media of aneurysmal walls 78. In contrast, Cystatin C was lowly expressed in the endothelial cell layer and the media of arterial wall of cerebral aneurysm 78. Increased expression of cathepsins and decreased expression of Cystatin C causes excessive degradation of extracellular matrix in the aneurysmal walls, which will lead to the progression and rupture of cerebral aneurysm 78, 80, 81. Treatment with NC‐2300, a selective inhibitor for cysteine cathepsins, decreased the activity of cathepsin B, K and S, inhibited the degradation of extracellular matrix in aneurysmal walls and prevented the progression of cerebral aneurysms 78. It is noteworthy that research on the role of cathepsins in the progression of cerebral aneurysms is still limited, and further investigations are anticipated, which may reveal new therapeutic avenues in preventing aneurysmal progression and rupture.

Taken together, accumulating lines of evidence indicate that the autophagy–lysosomal system is deeply involved in the pathophysiology of SAH. Thus, pharmacological modulation of the autophagy–lysosomal system may represent a potential therapeutic strategy to limit brain injury after SAH. Currently, several pharmacological agents that are able to modulate the autophagy–lysosomal system have been identified, such as mTOR inhibitors, AMPK modulators, calcium lowering agents and lysosome inhibitors 82, 83, 84. These modulators of the autophagy–lysosomal system could be tested in the treatment of SAH in future, many of whom appear to have high potential to be efficient.

## Perspective

Subarachnoid haemorrhage is a complex, multifaceted event that involves multiple ongoing processes contributing to its final pathogenesis. Despite great advances have been made in diagnostic methods, surgical and endovascular repair of ruptured aneurysms and management of medical complications, outcome for patients with SAH remains poor. Early brain injury and DBI, two major pathological mechanisms, are recognized as dominant contributors to the prognosis of SAH. The autophagy–lysosomal system is activated and plays a role in the pathogenesis of EBI and CVS after SAH (Table 1). It is significant to note that proper functioning of autophagy–lysosomal pathway acts as a pro‐survival mechanism to combat apoptotic cell death following SAH 56, 85. However, if SAH‐induced stress gets too high to deal with, lysosomal membranes would become destabilized so that hydrolytic enzymes would escape into the cytosol to trigger apoptotic cell death 70. Consequently, it is imperative to maintain the most appropriate threshold of autophagic activity for neuronal survival in the context of SAH, which would be beneficial for patient outcome after SAH.

**Table 1:** Main findings of the autophagy–lysosomal system in the pathogenesis of subarachnoid haemorrhage

Model	Stage	Main findings	Reference
Modified endovascular perforation rat model	EBI	Activation of autophagy–lysosomal pathway	Lee *et al*., 15
Prechiasmatic blood injection rat model	EBI	Activation of autophagy–lysosomal pathway Inhibition of EBI	Wang *et al*., 59
Endovascular perforation rat model	EBI	Activation of autophagy–lysosomal pathway Inhibition of EBI Anti‐apoptotic effect	Jing *et al*., 52
Endovascular perforation rat model	EBI	Activation of autophagy–lysosomal pathway Inhibition of EBI Anti‐apoptotic effect	Zhao *et al*., 53
Endovascular perforation rat model	EBI	Melatonin‐induced autophagy activation Inhibition of EBI Anti‐apoptotic effect	Chen *et al*., 55
Prechiasmatic blood injection rat model	EBI	Cystatin C‐induced autophagy activation Inhibition of EBI	Liu *et al*., 60
Endovascular perforation rat model	EBI	Endoplasmic reticulum stress‐induced autophagy activation Inhibition of EBI Anti‐apoptotic effect	Yan *et al*., 58
Endovascular perforation rat model	EBI	Trichostatin A‐induced autophagy activation Inhibition of EBI Anti‐apoptotic effect	Shao *et al*., 56
Cisterna magna blood injection rat model	CVS	Cystatin C‐induced autophagy activation Inhibition of CVS	Liu *et al*., 63
Prechiasmatic blood injection rat model	EBI	Deferoxamine‐mediated protection of lysosomal membrane Decreased release of cathepsin B/D Inhibition of EBI Anti‐apoptotic effect	Yu *et al*., 70
Prechiasmatic blood injection rat model 3	EBI	α‐Lipoic acid‐plus‐mediated chelation of intralysosomal iron Decreased release of cathepsin B/D Inhibition of EBI Anti‐apoptotic effect	Wang *et al*., 75

It is noteworthy that even though knowledge of autophagy–lysosomal system in SAH pathology, the precise roles and underlying mechanisms of autophagy–lysosomal pathway in the setting of SAH remain vague. Indeed, ‘self‐eating’ autophagy and ‘self‐killing’ apoptosis crosstalk with each other extensively in the pathophysiological conditions 86. Core directors, such as Beclin‐1, caspase family proteases and p53, play a crucial role in directing molecular switches between these two intimately connected processes 85, 87. Therefore, future investigating the role of those core directors will help to elucidate the interrelationship between autophagy and apoptosis in the setting of SAH. In addition, autophagy extensively communicates with other subtype of autophagy (*i.e*. CMA), as well as the ubiquitin‐proteasome system during the protein degradation process 88. Also, autophagy is intricately interlinked with necroptotic cell death 89. It has been shown that autophagy can either promote or suppress necroptosis under certain conditions 90, 91. However, the crosstalk between autophagy and necroptosis in SAH pathology remains largely unclarified. And a better knowledge of the interconnection between these degradation pathways is of great significance, with the goal of developing effective strategies to manipulate them for optimizing the therapeutic approaches for SAH.

## Conclusion

The autophagy–lysosomal system exerts critical roles in maintaining intracellular homeostasis in the brain under SAH conditions. Appropriate autophagy functions as protective mechanisms for cell survival after SAH, while excessive ‘self‐eating’ autophagy may lead to cell death. Therefore, approaching molecular mechanisms of autophagy–lysosomal system in the setting of SAH is anticipated, which may ultimately allow to develop effective therapeutic strategies for SAH patients through regulating this pathway.

## Conflict of interest

No potential conflicts of interest were disclosed.
